# Circulating microRNAs as Diagnostic Biomarkers to Detect Specific Stages of Ovarian Cancer: A Comprehensive Meta-Analysis

**DOI:** 10.3390/cancers16244190

**Published:** 2024-12-16

**Authors:** Apriliana Ellya Ratna Kartikasari, Paul Michel-Lara, Hayden Exton, Kaan Tekin-Sari, Ebtesam Motlaq M. Alnefai, Arnan Mitchell, Cesar Sanchez-Huertas, Magdalena Plebanski

**Affiliations:** 1 Cancer, Ageing, and Vaccine Research Group (CAVA), School of Health and Biomedical Sciences, RMIT University, Bundoora 3083, Australiared_rose-1212@hotmail.com (E.M.M.A.); 2 Integrated Photonics and Applications Centre (InPAC), School of Engineering, RMIT University, Melbourne 3001, Australia

**Keywords:** ovarian cancer, microRNAs, circulating biomarker, diagnosis, personalized therapy

## Abstract

Background: Ovarian cancer (OC) remains the deadliest gynecological malignancy, despite significant treatment advances. The absence of early detection tests results in advanced-stage diagnosis for 70% of OC cases. MicroRNAs (miRNAs) are known to be dysregulated in OC. Their presence or absence in circulation may indicate malignancy, potentially before tumor formation or metastasis. We hypothesize that miRNA expression patterns shift with OC progression and could facilitate stage-specific detection. Methods: We searched Pubmed, Embase, and Web of Science, and a total of 50 articles with 209 studies and 11,158 participants were included. We assessed the utility of circulating miRNAs for detecting OC, particularly at different stages, using the bivariate hierarchical random-effects model. Results: Using this model, the panels of miRNAs (83.92% sensitivity and 89.82% specificity) performed significantly better than single miRNAs (76.91% sensitivity and 70.56% specificity). Combining miRNA panels with the classical biomarkers, cancer antigen-125 and/or human epididymis protein significantly improved the detection to 93.39% sensitivity and 92.71% specificity. miR-200c, miR-1246, miR-141, miR-200b, miR-200a, and miR-429 are among the miRNA species with sensitivity and specificity greater than 70%. miR-141, miR-320b, miR-223, and miR-200c were frequently used to identify early-stage OC, showing 84.78% sensitivity and 82.37% specificity. For late-stage OC, miR-200c, miR141, miR-1246, and miR-320b were the frequent miRNAs used, providing 79.97% sensitivity and 81.97% specificity. While patient demographics and detection methods influence the variability in the diagnostic accuracy of miRNAs, factors such as the control types (healthy individuals or benign growths), the directions of level changes (upregulated and downregulated), and the sample sizes did not impact the diagnostic precision. Conclusion: This study highlights the utility of circulating miRNAs for OC detection at specific stages, enabling the development of minimally invasive early detection tests and allowing stage-specific approaches for more personalized and precise therapeutic management.

## Simple Summary

Currently, ovarian cancer is almost always diagnosed at its advanced stages, and it is the most lethal gynecological cancer amongst women. This study identifies the utility of miRNAs circulating in the blood to detect ovarian cancer at specific stages. This study highlights the use of circulating miRNAs not only for early detection of ovarian cancer but also to help enable more personalized and precise therapeutic management based on the identification of the stage of the cancer in a minimally invasive way, as only blood is required for their detection.

## 1. Introduction

Ovarian cancer (OC) is one of the most common gynecological cancers [1,2] and the eighth leading cause of cancer-related death in women globally [3]. The International Federation of Gynecology and Obstetrics (FIGO) categorizes ovarian cancer into four stages. Stages I and II are considered early stages, with the tumor localized within the ovaries and pelvic area, while stages III and IV are advanced stages, where the disease progresses from peritoneal metastasis to distant metastasis [4]. The progression of the disease to the advanced stages dramatically impacts survival rates. While early-stage OC diagnosis leads to a 70–90% survival rate, this figure plummets to a mere 20–40% for those diagnosed at stages III or IV [5]. Unfortunately, most women are frequently diagnosed at advanced stages [1,2]. This late detection of OC could be attributed to non-specific symptoms, as well as the absence of approved screening tests.

Conventional diagnostic approaches for ovarian cancer using transvaginal ultrasonography and serum levels of cancer antigen-125 (CA-125) or/and human epididymis protein (HE-4), the protein-based biomarkers for OC, have not been shown to reduce its mortality [6]. These methods are also inaccurate and can induce stress in patients [5]. While transvaginal ultrasonography can identify size and morphology changes in the ovary, it is unspecific for OC [5]. CA-125 levels can be elevated due to other conditions including benign gynecologic conditions, menopausal status, abdominal inflammation, and cardiac conditions [7,8,9,10]. Similarly, HE4 levels can be elevated in kidney diseases [11]. Such inaccuracy is particularly detrimental for detecting early-stage OC when the tumor burden is still low. Therefore, identifying other biomarkers that could help detect OC at its earliest stages is highly desired.

Various genetic and epigenetic mutations involving oncogenes or tumor suppressor genes [12] within normal cells can transform non-malignant cells into malignant cells, promoting cancer formation. Malignant cells proliferate regardless of the presence of growth or inhibitory signals, evade apoptotic pathways, and override intrinsic replication limits [13]. The primary tumor then induces angiogenesis to acquire the necessary nutrients and oxygen for its growth and to further metastasize to other parts of the body [12,13]. MicroRNAs (miRNAs), single-strand non-coding RNA sequences with an average length of only 18–22 bases, are involved in various stages of cancer development and progression [14]. miRNAs can have oncogenic or tumor suppressor functions by targeting genes involved in cancer processes [15]. These molecules downregulate gene expression at the post-transcriptional levels by binding to messenger RNAs (mRNAs) in the cytoplasm, promoting translational inhibition or mRNA degradation [16]. Conversely, other miRNAs can upregulate gene expression by directly targeting gene promoters [17] or inducing mRNA translation [18]. Intracellular miRNAs could be secreted into the extracellular space in the circulation by apoptotic or necrotic cells or actively released by cells in micro-vesicles as an intercellular communicator [19]. Regardless of their origin, due to their stability, circulating cell-free miRNAs have emerged as a new class of biomarkers with the potential to be used as a minimally invasive diagnostic indicator for the presence of cancer. Changes in cell-free miRNA levels in the circulation indeed have been suggested to indicate the presence of OC [20,21]. Previous meta-analyses have also correlated circulating cell-free miRNAs levels with the presence of OC [20,22]. However, their diagnostic accuracy, which specifically measures the potential of biomarkers to detect ovarian cancer, especially in its early stages, remains unknown.

This systematic review was conducted to synthesize and analyze the diagnostic potential of miRNAs, with a special focus on their accuracy when detecting specific stages of OC, as we hypothesize that miRNA expression patterns shift with OC progression and could facilitate stage-specific detection. This study was also conducted to identify miRNA species with strong potential to detect the presence of OC, important at early stages. We additionally analyzed the accuracy of circulating miRNAs to discriminate OC not only from the healthy controls but also from the benign growth, as OC and benign growths may have similar miRNA expression profiles. Uncovering the potential of circulating miRNAs for OC detection at specific stages will not only enable the development of early detection tests but also help in the development of stage-specific approaches for more personalized and accurate therapeutic management.

## 2. Materials and Methods

### 2.1. Study Design

This study aimed to evaluate information from published articles with meta-analysis on the evidence of the diagnostic utility of circulating miRNAs to detect OC, alone or in combination with the established circulating biomarkers, such as CA125 and HE4. A literature search was carried out by the authors using authentic scientific databases following the guidelines set by Preferred Reporting Items for Systematic Reviews and Meta-Analysis (PRISMA) [23].

### 2.2. Search Strategy

The literature was retrospectively searched using Pubmed, Embase, and Web of Science from inception to October 2024 for studies investigating circulating microRNA to detect OC. Our search strategy used the key words and designed strategy inputed into both databases as followed: (MiRNA OR microRNA) AND (ovary OR ovarian) AND (cancer OR cancers OR carcinoma OR tumor OR neoplasm malignant OR malignancy). The resulting literature was exported into EndNote citation manager, where duplicates were removed by EndNote. Titles and abstracts were then manually reviewed by five authors (AK, PL, HE, KT, and EA). An additional search by scanning reference lists from related articles was also performed. Articles that only used animal models or in vitro or ex vivo approaches are excluded. Case studies, previous meta-analyses, reviews, conference papers and articles that are yet to be peer-reviewed were excluded. The inclusion criteria included articles that are (a) on OC detection, (b) involving circulating miRNAs, (c) peer-reviewed, and (d) published as full-length articles. Then, articles were further selected based on their content relevant to this study.

### 2.3. Quality Assessment

To assess the quality of the selected articles, the Newcastle–Ottawa Scale (NOS) was used [24]. NOS assesses the quality of nonrandomized articles for meta-analyses. The article is judged based on three broad perspectives: (a) the selection of the study groups, (b) the comparability of the groups, and (c) the ascertainment of the exposure or the outcome. Each star was awarded when the articles fulfilled NOS criteria. A total of eight stars could be received by an article. Articles that received six or more stars were considered “good” or “quality”.

### 2.4. Data Extraction

Five authors (AK, PL, HE, KT, and EA) recorded data from all articles that met inclusion criteria using a standardized data collection procedure (Table 1). Extracted data from the selected articles include the title of the study, authorship, journal name, year, country of the study, study type, miRNA species, method of analyses, level changes, sample types, age of the participants, ethnicity, the total number of participants, control types (healthy participants or benign growth), and FIGO stages (Table S1). Stages I and II were considered early stages, while stages III and IV were considered late stages, as most articles reported combined data. Diagnostic parameters including specificity and sensitivity were extracted from either the provided measurements or calculated from the original data. Many articles reported the data based on multiple individual microRNAs, combinations of microRNAs, and/or panels of protein-based biomarkers in combination with microRNAs. In this case, each set of specificity and sensitivity values was treated as a study data point within the respective meta-analysis.

### 2.5. Data Analysis and Statistics

The changes in miRNA levels in various OC stages in comparison to the controls were mapped to improve the understanding of the role of specific miRNA species in OC development. The data were effectively separated into 5 groups: healthy, benign, early-stage OC (stage I and II), late-stage OC (stage III and IV), and all-stage OC. We back-calculated the true positive (FP), true negative (TN), false positive (FP), and false negative (FN) values on the basis of sample sizes [25]; then, the sensitivity TP/(TP + FN) and specificity TN/(FP + TN) were calculated. The overall sensitivity and specificity values, as well as odd ratios, were calculated using the bivariate random effect model at 95% CI. The bivariate random effects and hierarchical summary receiver operating characteristics (SROCs) that have been recommended for meta-analyses of diagnostic test accuracy studies [26] were generated to synthesize and evaluate the accuracy of miRNAs as diagnostic biomarkers for OC. Between-study heterogeneity was measured with Q and I^2^ tests. The sources of heterogeneity variance were tested using sub-grouping and meta-regression analysis. All statistical analyses were carried out using OpenMeta(analyst)^®^V.12.11.14 (Brown University, Providence, RI, USA) [27] and JAMOVI (version 2.5) by The Jamovi Project (2024), Sydney, Australia.

**Table 1:** Characteristics of the included articles.

No.	Article	Year	Country	No. of Participants of the Analyzed Studies	Discovery or a Priori Knowledge	miRNAs	Sample Type
1.	Kan et al. [28]	2012	Australia	56	a priori	miR-200a miR-200b miR-200c	Serum
2.	Zheng et al. [29]	2013	China	250	a priori	miR-205 let-7f	Plasma
3.	Suryawanshi et al. [30]	2013	USA	75	discovery	miR-16 miR-21 miR-191	Plasma
4.	Guo et al. [31]	2013	China	100	a priori	miR-92	Serum
5.	Gao and Wu [32]	2015	China	143	a priori	miR-200c miR-141	Serum
6.	Meng et al. [33]	2015	Germany	246	a priori	miR-7 miR-429 miR-25 miR-93	Serum
7.	Liang et al. [34]	2015	China	280	discovery	miR-145	Serum
8.	Zuberi et al. [35]	2015	India	140	a priori	miR-200a miR-200b miR-200c	Serum
9.	Meng et al. [36]	2016a	Germany	80	a priori	miR-200a miR-200b miR-200c	Serum
10.	Meng et al. [37]	2016b	Germany	183	a priori	miR-200a miR-200b miR-200c	Serum
11.	Zuberi et al. [38]	2016a	India	140	a priori	miR-199a	Serum
12.	Zuberi et al. [39]	2016b	India	140	a priori	miR-125b	Serum
13.	Zhu et al. [40]	2017	China	189	a priori	miR-125b	Serum
14.	Todeschini et al. [41]	2017	Italy	233	discovery	miR-1246 miR-595 miR-2278	Serum
15.	Yokoi et al. [42]	2017	Japan	269	discovery	miR-200a miR-766 miR-26a miR-142 let-7d miR-130b miR-328 mir374a	Serum
16.	Elias et al. [43]	2017	United States	211	discovery	miR-29a miR-92a miR-200c miR-320c miR-335 miR-450b miR-1307	Serum
17.	Kobayashi et al. [44]	2018	Japan	70	a priori	miR-1290	Serum
18.	Pan et al. [45]	2018	Germany	135	a priori	miR-21 miR-100 miR-200b miR-320	Plasma
19.	Yokoi et al. [5]	2018	Japan	530	discovery	miR-663b miR-4730 miR-642a miR-658 miR-486 miR-1246 miR-1207 miR-4419b miR-6124	Serum
20.	Yoshimura et al. [46]	2018	Japan	108	a priori	miR-99a	Serum
21.	Ren et al. [47]	2018	China	160	a priori	miR-193a	Serum
22.	Mahmoud et al. [48]	2018	Egypt	90	a priori	miR-21	Serum
23.	Kim et al. [49]	2019	Korea	68	a priori	miR-145 miR-200c	Serum
24.	Oliviera et al. [50]	2019	Denmark	190	a priori	miR-200c miR-221	Serum
25.	Wang et al. [51]	2019	China	160	discovery	miR-200c miR-346 miR-127 miR-143 miR-205	Serum
26.	Márton et al. [52]	2019	Hungary	100	a priori	miR-200a miR-200b miR-200c miR-141 miR-429 miR-203a miR-34a miR-34b	Plasma
27.	El-Shal et al. [53]	2019	Egypt	137	a priori	miR-361	Serum
28.	Liang et al. [54]	2020	China	151	a priori	miR-183 miR-182 miR-205	Serum
29.	Chen et al. [55]	2020a	China	104	a priori	miR-152	Serum
30.	Chen et al. [56]	2020b	China	301	a priori	miR-125b	Serum
31.	Zuberi et al. [57]	2020	India	140	a priori	miR-145	Serum
32.	Cirillo et al. [58]	2021	Italy	177	discovery	miR-320b miR-141	Serum
33.	Kumar et al. [59]	2021	India	65	a priori	miR-205 miR-200c miR-141	Serum
34.	Hashimoto et al. [60]	2021	Japan	20	discovery	miR-1307	Serum
35.	Jeon et al. [61]	2022	Korea	84	a priori	miR-1290	Serum
36.	Zhu et al. [62]	2022	China	68	a priori	miR-205	Plasma
37.	Hannan et al. [63]	2022	Australia	77	discovery	miR-200c	Plasma
38.	Ali et al. [64]	2022	Egypt	120	a priori	miR-204	Serum
39.	Zhang and Hu [65]	2022	USA	3092	discovery	miR-5100 miR-1343 miR-1290 miR-4787	Serum
40.	Wang et al. [66]	2022	China	363	a priori	miR-320d miR-4479 miR-6763	Plasma
41.	Chen et al. [67]	2022	China	283	a priori	miR-1260a miR-7977 miR-192	Plasma
42.	Raiser et al. [68]	2022	Germany	76	a priori	miR-21 miR-1	Serum
43.	Gahlawat et al. [69]	2023	Germany	125	a priori	miR-200c miR-141	Plasma
44.	Niemira et al. [70]	2023	Poland	728	discovery	miR-1246 miR-150 miR-144	Serum
45.	Takamizawa et al. [71]	2024	Japan	47	a priori	miR-146a miR-191	Serum
46.	Li et al. [72]	2023	China	79	discovery	miR-1246 miR-141 miR-200a miR-200b miR-200c miR-203a miR-429	Serum
47.	Gumusoglu-Acar et al. [73]	2023	Turkey	20	a priori	miR-1909 miR-885 let-7d	Serum
48.	Yang et al. [74]	2024	China	170	a priori	miR-2234	Plasma
49.	Minareci et al. [75]	2024	Turkey	135	a priori	miR-4314 miR-1181	Serum
50.	Tuncer et al. [76]	2024	Turkey	250	a priori	miR-3135b miR-1273g	Blood

## 3. Results

### 3.1. Identification and Characteristics of the Selected Articles

**Figure 1:**
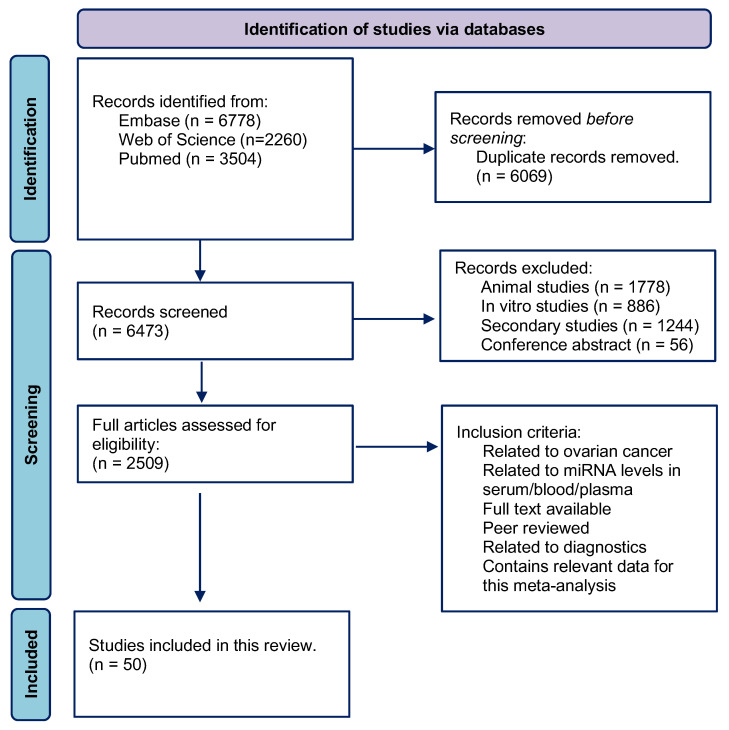
PRISMA flowchart of the systematic review. The search method identified relevant articles from three databases. After duplicates were removed, articles were screened against inclusion and exclusion criteria. The resulting articles (*n* = 50) were further analyzed for the subsequent meta-analysis.

We followed a PRISMA flowchart to record the number of articles searched from Pubmed, Embase, and Web of Science (Figure 1). A total of 6473 articles were identified from these databases after the duplicates were removed from the selection. The content of the articles was then screened by five authors (AK, PL, HE, KT, and EA). We removed 1778 articles on animal studies, 886 in vitro and ex vivo studies, 1244 secondary articles, and 56 conference articles. After removing those articles, 2509 articles were analyzed for their relevance and data content following the inclusion criteria. The final 50 articles [5,28,29,30,31,32,33,34,35,36,37,38,39,40,41,42,43,44,45,46,47,48,49,50,51,52,53,54,55,56,57,58,59,60,61,62,63,64,65,66,67,68,69,70,71,72,73,74,75,76] involving 209 case–control studies were included in our data collection and meta-analysis (Table 1 and Table S1).

All included studies (Table 1) were retrospectively conducted. These articles analyzed circulating miRNAs extracted either from serum, plasma, or blood. There were two types of approaches conducted by the studies—the ones that involved a discovery experiment to determine the best miRNA candidates followed by a validation phase and the ones that selected miRNA candidates based on a priori knowledge (Table 1). Various assays including miRNA sequencing, miRNA microarray, quantitative PCR, and digital droplet PCR were used to measure the levels of the circulating miRNAs (Table S1). The selected articles were largely conducted in the Eastern Asian and European populations (Table 1). The age of the patients with cancer was generally reported to be well matched to the age of the control subjects; however, the sample sizes varied largely between studies (Table 1 and Table S1). Types of controls including healthy participants and benign growths were used (Table S1). The levels of the selected miRNAs used to discriminate OC from controls were mostly increased, while a few were decreased in patients with cancer compared to the controls (Table S1). Twenty-four case–control studies reported the diagnostic utility of miRNAs to specifically detect early-stage OC whilst thirty-two studies reported specific detection of late-stage OC (Table S1). A-hundred-and-forty studies reported the detection potential of a single miRNA, and sixty-nine studies reported the potential of a panel of several miRNAs or a combination of a panel of miRNAs with cancer-specific protein-based biomarkers such as CA125 and HE4 (Table S1).

We further assessed the quality of the selected articles using NOS, which was designed to assess nonrandomized studies in meta-analyses. All the included articles had a total score of 7 or more; thus, they were above the acceptable quality threshold (Table S2).

### 3.2. Diagnostic Accuracy of miRNAs for OC Detection

**Figure 2:**
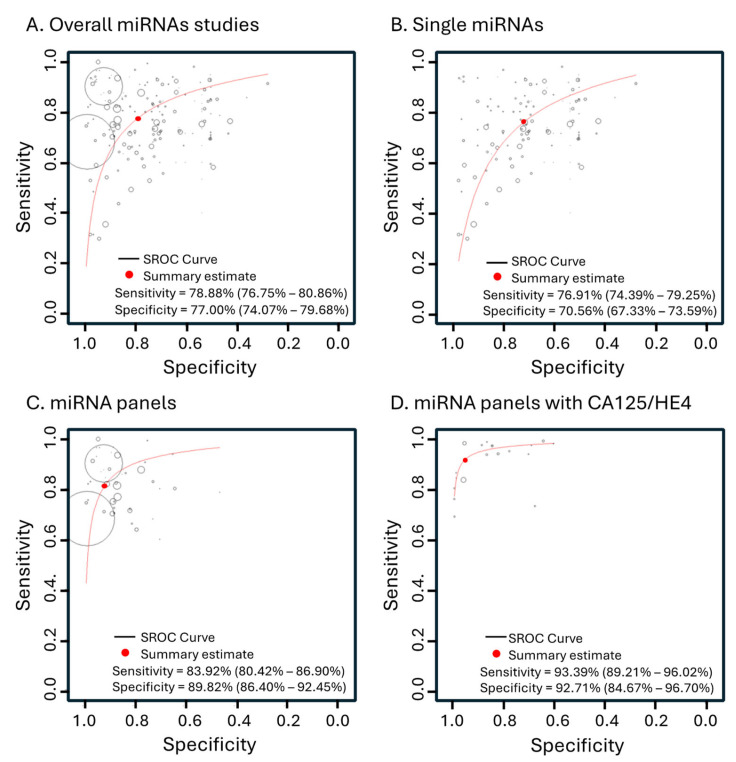
The hierarchical summary receiver operating characteristic curve (SROC) from overall miRNA studies and the subgroups. (**A**) Bivariate random effects analysis of 189 studies involving only circulating miRNAs to detect various stages of OC. (**B**) Subgroup analysis of 140 studies that used single miRNAs. (**C**) Subgroup analysis of 49 studies that used panels of miRNAs. (**D**) Subgroup analysis of 20 studies that used miRNA panels combined with CA125 and/or HE4.

We next extracted the data from each study that measured specificity and sensitivity of miRNAs of interest. We used the hierarchical method with bivariate random effects weighting to provide diagnostic accuracy summary, as this method also accounts for the correlation between sensitivity and specificity [26]. We then grouped the studies based on their specifications, such as the type of biomarkers and the type of samples used, among others. The overall accuracy summary of 189 studies involving only circulating cell-free miRNAs and not in combination with other type of biomarkers such as CA125 and/or HE4 to detect various stages of OC showed 78.88% sensitivity (95% confident interval (95% CI): 76.75–80.86%) and 77.00% specificity (95% CI: 74.07–79.68%), with an overall diagnostic odds ratio (DOR) of 11.384 (95% CI: 9.479–13.672) (Figure 2A), suggesting the potential of miRNAs to detect OC.

We further explored the diagnostic accuracy of miRNAs when used individually or in a group or panel to predict OC. To do this, we categorized the studies into two groups: those using a single miRNA and those using a panel of miRNAs. The studies that only used single miRNAs to detect OC showed suboptimal accuracy, with 76.91% sensitivity (95% CI: 74.39–79.25%), 70.56% specificity (95% CI: 67.33–73.59%), and a DOR of 7.644 (95% CI: 6.554–8.915). The use of multiple miRNAs showed higher overall accuracy (*p* < 0.0001), with 83.92% sensitivity (95% CI: 80.42–86.90%) and 89.82% specificity (95% CI: 86.40–92.45%) and a DOR of 37.054 (95% CI: 25.146–54.601) (Figure 2B,C, Table 2). Thus, this finding suggests that multiple miRNAs provide better diagnostic accuracy in comparison to single miRNAs.

### 3.3. Diagnostic Accuracy of miRNAs when Combined with the Classical Biomarkers CA125 and HE4

**Figure 3:**
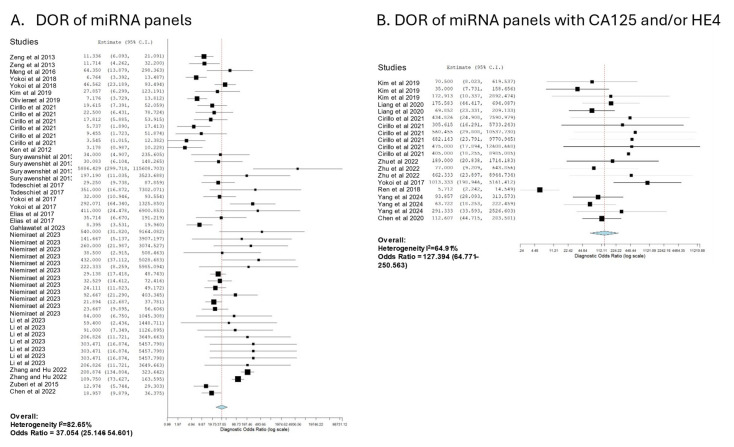
Forest plots of the diagnostic odds ratio (DOR) generated by the bivariate random effect analysis of (**A**) 49 studies that used panels of miRNAs and (**B**) 20 studies that used miRNA panels combined with CA125 and/or HE4. Forest plots display the effects of each individual study included in the meta-analysis. The size of the square indicates the sample size, while the measured value is shown on the x-axis, and the confidence interval is represented by the horizontal line. The blue diamond reflects the calculated overall measured value, based on a random-effects model.

Since CA125 and HE4 are consistently upregulated in OC patients, we also analyzed 20 studies that have used miRNA panels in combination with these protein biomarkers to indicate the presence of OC. The miRNA panels combined with CA125 and/or HE4 showed a sensitivity of 93.39% (95% CI: 89.21–96.02%) and specificity of 92.71% (95% CI: 84.67–96.70%), with a diagnostic odds ratio (DOR) of 127.394 (95% CI: 64.771–250.563) (Figure 2D and Figure 3). This represents a significant improvement in comparison to miRNA panels alone (*p* < 0.0001), indicating that incorporating CA125 and/or HE4 further enhances the diagnostic accuracy of miRNAs.

### 3.4. Subgroup Contributions to Accuracy and Heterogeneity

**Figure 4:**
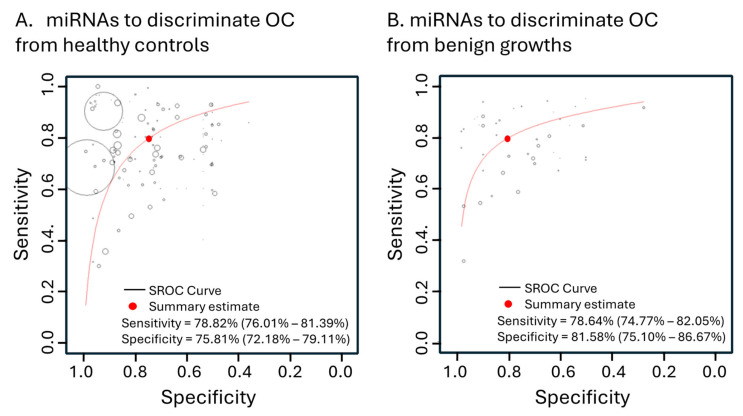
Hierarchical summary receiver operating characteristic curve (SROC) from the miRNA studies using (**A**) healthy controls (127 studies) and (**B**) benign growths (48 studies).

Due to the high heterogeneity found among the pooled 189 miRNA studies (I^2^ of 82.04%, Table 2), we explored subgrouping those studies based on their characteristics and conducted a meta-regression analysis. This approach aimed to enhance our understanding of what factors influence diagnostic accuracy and heterogeneity between studies. As shown in Table 2 and consistent with the results shown above, the choice of using multiple miRNAs over single miRNAs significantly contributed to a better overall diagnostic accuracy of miRNA studies (*p* < 0.0001). The demographics of the participants (*p* < 0.0001), as well as the method of detection used (*p* = 0.0024), significantly affected the diagnostic accuracy and heterogeneity (Table 2). On the other hand, the type of samples, whether serum or plasma, contributed marginally to overall diagnostic accuracy and heterogeneity (*p* = 0.0016, Table 2). Furthermore, OC stages, number of participants, the direction of changes in the circulating miRNA levels, and, more importantly, types of control population, i.e., healthy or benign growths, did not affect the heterogeneity and diagnostic accuracy of the pooled miRNA studies (Table 2). The miRNA studies that used healthy controls showed overall 78.82% sensitivity (95% CI: 76.01–81.39%), 75.81% specificity (95% CI: 72.18–79.11%), and 10.708 DOR (95% CI: 8.474–13.531), which was not significantly different (*p* = 0.4592) when benign controls were used, showing 78.64% sensitivity (95% CI: 74.77–82.05%), 81.58% specificity (95% CI: 75.10–86.67%), and a DOR of 12.365 (95% CI: 9.152–16.704) (Table 2, Figure 4).

### 3.5. Specific miRNA Panels Able to Detect Different Stages of OC Cases

**Figure 5:**
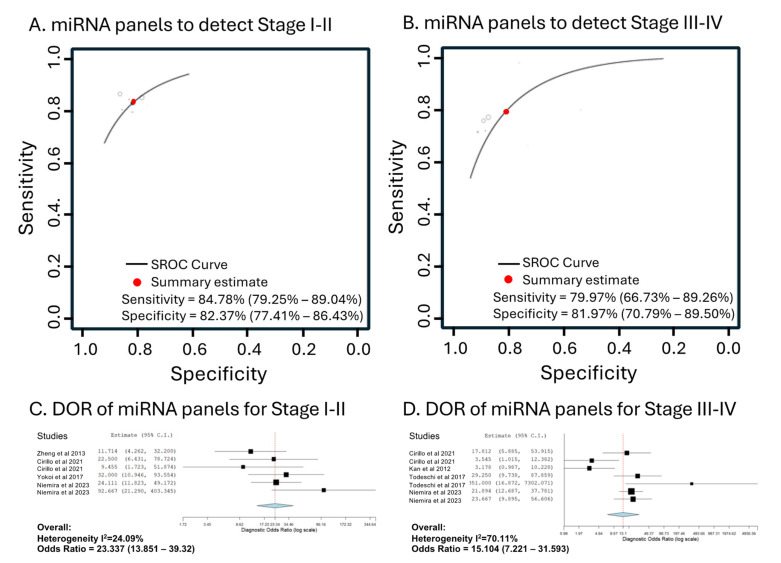
Hierarchical summary receiver operating characteristic curve (SROC) from the miRNA panels to detect (**A**) stages I–II with 6 studies and (**B**) stages III–IV with 7 studies; (**C**,**D**) show corresponding forest plots of the diagnostic odds ratio (DOR). The size of the square indicates the sample size, while the measured value is shown on the *x*-axis, and the confidence interval is represented by the horizontal line. The blue diamond reflects the calculated overall measured value, based on a random-effects model.

We further analyzed the overall diagnostic accuracy of miRNAs specifically detecting either early stages (stages I and II) or late stages (stages III and IV). As expected, for this stage-specific detection, using multiple miRNAs outperformed single miRNAs (Table 3). The types of control population and the direction of changes in miRNA levels did not significantly influence the diagnostic accuracy in detecting OC at these specific stages (Table 3). Overall, the accuracy of the panel miRNAs for early-stage OC showed a sensitivity of 84.78% (95% CI: 79.25–89.04%), a specificity of 82.37% (95% CI: 77.41–86.43%), and a DOR of 23.337 (95% CI: 13.85–39.320) comparable to that of late-stage OC, with a sensitivity of 79.97% (95% CI: 66.73–89.26%), a specificity of 81.97% (95% CI: 70.79–89.50%), and a DOR of 15.104 (95% CI: 7.221–31.593) (Table 3, Figure 5).

### 3.6. Best Individual miRNAs to Detect Early- and Late-Stage OC

**Figure 6:**
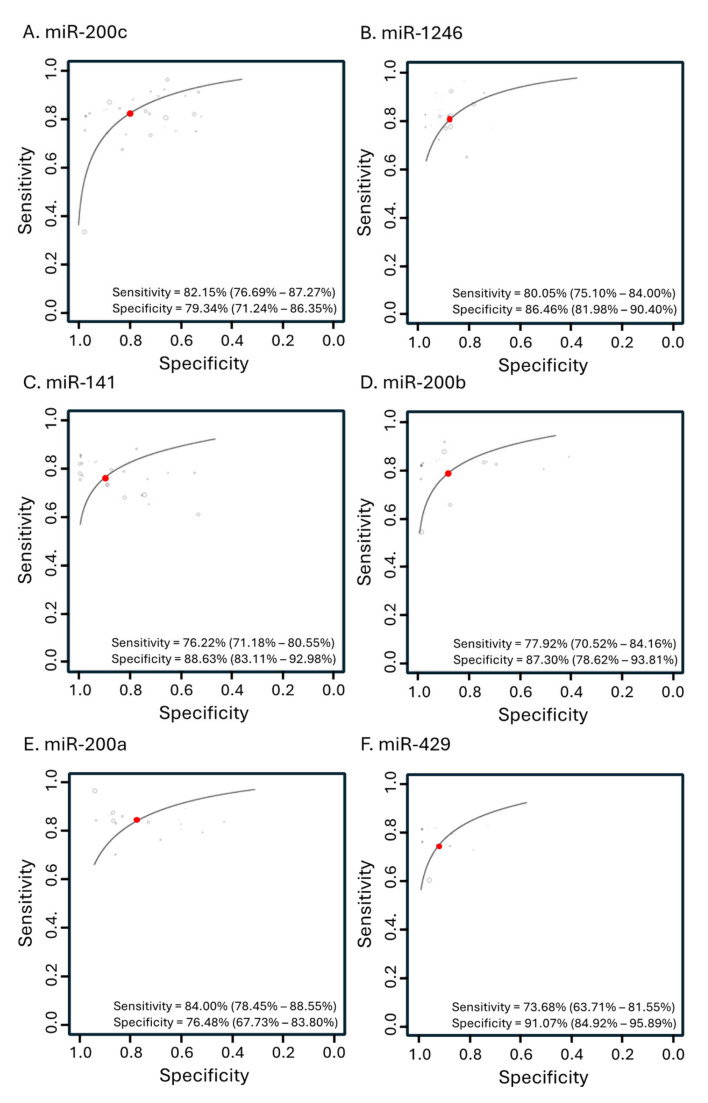
Hierarchical summary receiver operating characteristic curve (SROC) of the most frequent miRNAs being used to detect OC in all stages. (**A**) miR-200c with 30 studies, (**B**) miR-1246 with 27 studies, (**C**) miR-141 with 25 studies, (**D**) miR-200b with 17 studies, (**E**) miR-200a with 16 studies, and (**F**) miR-429 with 13 studies.

We further analyzed how often individual miRNAs were used to identify specific stages of OC. Overall, there were 24 studies specifically involving stages I–II and 32 studies specifically involving stages III–IV. Table 4 shows the list of the individual miRNAs and their frequency to appear in studies. miR-141, miR-320b, miR-223, and miR-200c are among the miRNAs that are frequently selected to detect OC stages I–II, while miR-200c, miR-141, miR-1246, and miR-595 are frequently used to detect OC stages III–IV (Table 4). The direction of changes in miRNA levels in individuals with OC compared to combined healthy and benign controls are also shown (Table 4). Overall, the miRNA200 family frequently appeared in the studies detecting various stages of OC. The diagnostic accuracy analyses of these individual miRNAs indeed showed their promising potential to aid the detection of OC, with overall sensitivity and specificity >70% (Figure 6).

## 4. Discussion

Extracellular circulating miRNAs are short non-coding RNAs comprising only 20–25 nucleotides and are remarkably stable [77]. They are derived during passive release from apoptotic and necrotic cells or can be actively released as RNA–protein complexes or within lipoproteins and exosomes to signal other cells [78]. More than 2500 miRNAs have been mapped in the human genome [79], each exhibiting unique expression patterns and functions in various types of cancers. Indeed, specific miRNAs have been correlated with the initiation, development, invasion, and metastasis of different cancers [80], suggesting their potential as biomarkers to differentiate cancer stages when found in circulation. This potential is particularly important in OC, where early detection is crucial. In gynecological cancers, several clinical trials to investigate the diagnostic accuracy of circulating cell-free miRNAs have recently been undertaken, for example, to detect multicentric breast cancer (ClinicalTrials.gov NCT04516330) or various gynecological cancers (ClinicalTrials.gov NCT03776630). Utilizing circulating cell-free miRNAs as biomarkers to accurately detect the presence of OC, especially at its earliest stage, could be a game changer for its diagnosis, offering minimally invasive, cost-effective, and accessible methods, improving survival rates.

In this meta-analysis, we showed that groups of miRNAs provide high diagnostic accuracy for OC detection, with a sensitivity of 83.92% and a specificity of 89.82%. The sensitivity and specificity reported in this study are significantly higher than those of the widely known OC biomarkers, namely CA25 and HE4, which are primarily used for monitoring recurrent OC. The findings here suggest that miRNAs could provide improved diagnostic accuracy in detecting OC in comparison to CA125 and HE4. When combined with CA125 and HE4, miRNAs achieved a sensitivity of 93.39% and a specificity of 92.71%, showing high accuracy as an approach to be used as diagnostic biomarkers. Subsequent group analysis showed that a panel of miRNA biomarkers provide better accuracy than using only a single miRNA species. Furthermore, the geography of the population contributes to the heterogeneity of the diagnostic accuracy, with Northern American studies showing the highest diagnostic accuracy. The selected method of detection further contributes to the diagnostic accuracy, with digital PCR providing the best accuracy. Types of samples, i.e., plasma or serum, only marginally showed differences in diagnostic accuracy, while types of controls, i.e., healthy or benign growths, sample sizes, and the directions of miRNA level changes did not affect the diagnostic accuracy. These findings underscore the potential of miRNAs to detect various stages of OC in clinical settings; however, the accuracy is highly dependent on the panel of miRNAs, the geography of the population, and the detection method of these miRNAs.

We observed relatively high diagnostic accuracies of miRNAs to detect early-stage and late-stage OCs, with sensitivities/specificities of 84.78%/82.37%, and 79.97%/81.97%, respectively. Each stage showed unique changes in miRNA patterns and could be employed to stratify OC patients based on their stage of development. In this meta-analysis, we observed that the most frequently used miRNAs in all stages with various panels of miRNAs, geographical populations, and methods of detection are miR-200c, miR-1246, miR-141, miR-200b, miR-200a, and miR-429, with a sensitivity and specificity, individually, of more than 70%. These miRNAs may serve as the best biomarkers for OC in general. Yokoi et al. [42] identified a specific miRNA panel with 86% sensitivity and 83% specificity for detecting early-stage OC in the East Asian population, while Niemira et al. [70] reported a different set of miRNAs with 87% sensitivity and 93% specificity for detecting OC in the European population. This emphasizes the fact that, while a general miRNA panel could be effective across different populations and stages of OC, the results also suggest that the most effective miRNA panel may vary based on the geographical factors specific to each population.

The key limitation of this meta-analysis lies in the characteristics of the available studies, as the accuracy is dependent of the methods of detection, and the studies were predominantly focused on specific geographical populations, which may limit the generalizability of the findings to broader or more diverse populations. Future studies should aim to compare or tailor miRNA panels in distinct populations, as the dysregulated miRNAs may vary depending on the population measured. Additionally, there is a lack of studies specifically designed to test the utility of miRNAs to detect stage I OC, creating a gap in understanding their potential role at this initial stage. This absence presents an important opportunity for future research to explore and validate the diagnostic or prognostic value of miRNAs in stage I OC, which would ultimately enhance our ability to detect and manage the disease in its initial stages.

## 5. Conclusions

In summary, our meta-analysis demonstrates that panels of circulating miRNAs are promising minimally invasive biomarkers for detecting OC, including at its early stages, and can effectively distinguish between cancerous and benign growths. This meta-analysis shows that the selection of miRNA panels, the geography of the population, and the method of detection influence diagnosis accuracy. miR-200c, miR-1246, miR-141, miR-200b, miR-200a, and miR-429 are among miRNA species with a sensitivity and specificity greater than 70% in a variety of studies, making them worth testing for potential future clinical use. Furthermore, specific miRNA panels can identify distinct stages of the disease, enabling more personalized and precise treatment decisions. In summary, these findings suggest that miRNA panels could be integrated into routine practice for early detection and stage-specific management of ovarian cancer, pending further validation.

## Figures and Tables

**Table 2:** Summary estimates of the diagnostic accuracy of the miRNA biomarker subgroups using the bivariate random effects model.

Subgroup	No. of Studies	Sensitivity % (95% CI)	Specificity % (95% CI)	DOR (95% CI)	DOR (*p*-Value)	DOR I^2^	Subgroup Difference (*p*-Value)
All studies with miRNAs only as biomarkers							
	189	78.88 (76.75–80.86)	77.00 (74.07–79.68)	11.384 (9.479–13.672)	<0.001	82.036	
Number of miRNAs							
-Single	140	76.91 (74.39–79.25)	70.56 (67.33–73.59)	7.644 (6.554–8.915)	<0.001	65.294	<0.0001 *
-Panel	49	83.92 (80.42–86.90)	89.82 (86.40–92.45)	37.054 (25.146–54.601)	<0.001	82.646	
Sample type							
-Serum	146	81.24 (78.96–83.33)	77.85 (74.16–81.14)	13.505 (10.714–17.023)	<0.001	82.723	0.0016 *
-Plasma	41	70.15 (65.96–74.02)	76.10 (71.70–80.02)	7.755 (6.306–9.538)	<0.001	55.624	
Control type							
-Healthy	127	78.82 (76.01–81.39)	75.81 (72.18–79.11)	10.708 (8.474–13.531)	<0.001	85.185	0.7593
-Benign	48	78.64 (74.77–82.05)	81.58 (75.10–86.67)	12.365 (9.152–16.704)	<0.001	59.274	
Stages							
-Early: I–II	24	80.36 (75.91–84.16)	68.35 (60.75–75.08)	8.809 (5.440–14.264)	<0.001	72.359	0.4699
-Late: III–IV	32	79.81 (73.33–85.04)	71.25 (63.00–78.29)	9.190 (6.013–14.045)	<0.001	69.916	
No. of participants							
-Low (<100)	105	81.20 (78.74–83.43)	74.16 (70.00–77.93)	10.467 (8.278–13.235)	<0.001	59.665	0.7528
-High (>100)	84	76.05 (72.50–79.28)	79.68 (75.63–83.21)	11.944 (9.148–15.595)	<0.001	89.407	
Countries of the studies							
-East Asia	100	77.55 (74.97–79.93)	70.95 (66.75–74.81)	7.576 (6.336–9.057)	<0.001	63.535	<0.0001 *
-South Asia	11	83.16 (75.92–88.55)	83.51 (75.00–89.53)	22.973 (11.683–45.175)	<0.001	71.616	
-North America	6	87.49 (77.65–93.36)	93.76 (83.60–97.90)	99.166 (51.946–189.310)	<0.001	62.073	
-Europe	55	79.62 (74.46–83.97)	84.01 (80.50–86.99)	17.551 (12.970–23.752)	<0.001	73.452	
Methods used							
-Quantitative PCR	112	82.95 (79.95–85.58)	73.01 (69.29–76.43)	11.666 (9.413–14.457)	<0.001	72.379	0.0024 *
-Digital PCR	12	70.77 (61.99–77.36)	92.10 (85.37–96.67)	37.301 (14.846–93.721)	<0.001	70.396	
-Microarrays	10	69.63 (58.25–78.70)	86.21 (78.50–92.33)	12.396 (5.761–26.673)	<0.001	78.681	
-NGS	9	85.39 (76.53–91.59)	78.00 (66.79–86.69)	17.551 (12.970–23.752)	<0.001	0.001	
miRNAs levels							
-Upregulated	135	78.88 (76.44–81.13)	74.99 (71.33–78.32)	10.539 (8.394–13.233)	<0.001	80.136	0.2261
-Downregulated	35	74.02 (68.81–78.63)	75.26 (68.22–81.17)	8.161 (5.887–11.313)	<0.001	82.433	

* = *p*-values < 0.05, statististically significant for subgroup differences based on Q statistics.

**Table 3:** Summary estimates of the diagnostic accuracy of the stage-specific miRNA subgroups based on the bivariate random effects model.

Subgroup	No. of Studies	Sensitivity% (95%CI)	Specificity% (95% CI)	DOR (95% CI)	DOR (*p*-Value)	DOR I^2^	Subgroup Difference (*p*-Value)
Studies of Stage I–II OC with miRNAs as biomarkers							
-Overall	24	80.36 (75.91–84.16)	68.35 (60.75–75.08)	8.809 (5.440–14.264)	<0.001	72.359	
-Single miRNA	18	78.90 (73.32–83.58)	61.74 (53.21–69.59)	6.063 (3.675–10.069)	<0.001	63.054	0.0060 *
-Panel miRNA	6	84.78 (79.25–89.04)	82.37 (77.41–86.43)	23.337 (13.851–39.320)	<0.001	24.093	
-Against healthy controls	22	80.50 (75.65–84.58)	68.28 (60.29–75.31)	8.814 (5.355–14.507)	<0.001	70.398	>0.999
-Upregulated	17	79.00 (73.86–83.35)	65.56 (57.76–72.60)	7.178 (4.489–11.477)	<0.001	56.545	0.5292
-Downregulated	7	83.71 (74.07–90.23)	74.21 (57.01–86.19)	14.731 (4.659–46.581)	<0.001	84.507	
Studies of Stage III–IV OC with miRNAs as biomarkers							
-Overall	32	79.81 (73.33–85.04)	71.25 (63.00–78.29)	9.190 (6.013–14.045)	<0.001	69.916	
-Single miRNA	25	79.70 (72.13–85.62)	67.08 (57.51–75.41)	7.788 (4.739–12.800)	<0.001	66.629	0.3749
-Panel miRNA	7	79.97 (65.73–89.26)	81.97 (70.79–89.50)	15.104 (7.221–31.593)	<0.001	70.114	
-Against healthy controls	30	79.48 (72.64–84.97)	72.13 (63.46–79.41)	9.365 (6.009–14.596)	<0.001	71.376	>0.999
-Upregulated	29	79.63 (72.50–85.28)	67.18 (59.15–74.32)	7.517 (4.936–11.445)	<0.001	61.319	0.5122

* = *p*-values < 0.05, statististically significant for subgroup differences based on Q statistics.

**Table 4:** List of individual miRNAs that are being used in miRNA studies in specific stages of OC.

miRNAs in Stage I–II	Levels in Cancers vs. Healthy/ Benign Controls	No. of Studies	miRNAs in Stage III–IV	Levels in Cancers vs. Healthy/ Benign Controls	No. of Studies	miRNAs in All Stages	Levels in Cancers vs. Healthy/ Benign Controls	No. of Studies
miR-141	increased	6	miR-200c	increased	6	miR-200c	increased	30
miR-320b	increased	4	miR-141	increased	6	miR-1246	increased	27
miR-223	increased	3	miR-1246	increased	6	miR-141	increased	25
miR-200c	increased	2	miR-320b	increased	4	miR-200b	increased	17
miR-1246	increased	2	miR-595	increased	4	miR-200a	increased	16
miR-200a	increased	2	miR-2278	increased	4	miR-429	increased	13
miR-205	increased	2	miR-200a	increased	3	miR-320b	increased	12
miR-150	increased	2	miR-200b	increased	2	miR-205	increased	11
miR-142	increased	2	miR-150	decreased	2	miR-150	decreased	10
miR-328	increased	2	miR-205	increased	1	miR-145	decreased	9
miR-766	decreased	2	miR-145	decreased	1	miR-21	increased	5

## Data Availability

The original contributions presented in this study are included in the article/Supplementary Materials. Further inquiries can be directed to the corresponding authors.

## Supplementary Materials

The following supporting information can be downloaded at https://www.mdpi.com/article/10.3390/cancers16244190/s1, Table S1: Extracted data from the selected articles; Table S2: NOS assessment.
