# Supplementary material for: Circulating microRNAs as Diagnostic Biomarkers to Detect Specific Stages of Ovarian Cancer: A Comprehensive Meta-Analysis
Source: Cancers (Basel). 2024 Dec 16;16(24):4190. doi: 10.3390/cancers16244190 (PMC11674734; doi:10.3390/cancers16244190)
Supplement: Supplementary file 1 [file cancers-16-04190-s001.zip › Table S2.pdf]

Table S2. Newcastle-Ottawa quality scale of studies included in this meta-analysis study

**Source**	**Selection**				**Comparability**		**Exposure**			**Total score**
	1	2	3	4	5A	5B	6	7	8	
**Kan et al. 2012**	*	*	*	*	*	*	*	*	*	9
**Zheng et al. 2013**	*	*	*	*	*	*	*	*	*	9
**Suryawanshi et al. 2013**	*	*	*	*	*		*	*	*	8
**Guo et al. 2013**	*	*	*	*	*	*	*	*	*	9
**Gao and Wu. 2015**	*	*	*	*	*	*	*	*	*	9
**Meng et al. 2015**	*	*	*	*	*	*	*	*	*	9
**Liang et al. 2015**	*	*	*		*	*	*	*	*	8
**Zuberi et al. 2015**	*	*	*		*	*	*	*	*	8
**Meng et al. 2016a**	*	*	*	*	*		*	*	*	8
**Meng et al. 2016b**	*	*	*		*		*	*	*	7
**Zuberi et al 2016a**	*	*	*		*		*	*	*	7
**Zuberi et al 2016b**	*	*	*		*		*	*	*	7
**Zhu et al. 2017**	*	*	*	*	*	*	*	*	*	9
**Todeschini et al. 2017**	*	*	*	*	*	*	*	*	*	9
**Yokoi et al. 2016**	*	*	*		*	*	*	*	*	8
**Elias et al. 2017**	*	*	*	*	*	*	*	*	*	9
**Kobayashi et al. 2018**	*	*	*	*	*		*	*	*	8
**Pan et al. 2018**	*	*	*	*	*		*	*	*	9
**Yokoi et al. 2018**	*	*	*	*	*	*	*	*	*	9
**Yoshimura et al. 2018**	*	*	*	*	*		*	*	*	8
**Ren et al. 2018**	*	*	*	*	*	*	*	*	*	9
**Mahmoud et al. 2018**	*	*	*		*		*	*	*	7
**Kim et al. 2019**	*	*	*	*	*		*	*	*	8
**Oliviera et al. 2019**	*	*	*	*	*		*	*	*	8
**Wang et al. 2019**	*	*	*	*	*		*	*	*	8
**Marton et al. 2019**	*	*	*	*	*		*	*	*	8

**El-Shal et al. 2019**	*	*	*	*	*	*	*	*	*	9
**Liang et al. 2020**	*	*	*	*	*		*	*	*	8
**Chen et al. 2020a**	*	*	*	*	*	*	*	*	*	9
**Chen et al. 2020b**	*	*	*	*	*		*	*	*	8
**Zuberi et al. 2020**	*	*	*	*	*		*	*	*	8
**Cirillo et al. 2021**	*	*	*	*	*	*	*	*	*	9
**Kumar et al. 2021**	*	*	*	*	*		*	*	*	8
**Hashimoto et al.2021**	*	*	*	*	*		*	*	*	8
**Jeon et al. 2022**	*	*	*	*	*	*	*	*	*	9
**Zhu et al. 2022.**	*	*	*	*	*		*	*	*	8
**Hannan et al. 2022**	*	*	*	*	*		*	*	*	8
**Ali et al. 2022**	*	*	*	*	*		*	*	*	8
**Zhang and Hu 2022**	*	*	*	*	*		*	*	*	8
**Wang et al. 2022**	*	*	*	*	*		*	*	*	8
**Chen et al. 2022**	*	*	*	*	*	*	*	*	*	9
**Raiser et al. 2022**	*	*	*	*	*	*	*	*	*	9
**Gahlawat et al. 2023**	*	*	*	*	*	*	*	*	*	9
**Niemira et al. 2023**	*	*	*	*	*		*	*	*	8
**Takamizawa et al. 2024**	*	*	*	*	*	*	*	*	*	9
**Li et al. 2023**	*	*	*	*	*		*	*	*	8
**Gumusoglu-Acar et al. 2023**	*	*	*	*	*		*	*	*	8
**Yang et al. 2024**	*	*	*	*	*		*	*	*	8
**Minareci et al 2024**	*	*	*	*	*	*	*	*	*	9
**Tuncer et al. 2024**	*	*	*		*		*	*	*	7
