# Supplementary material for: Circulating microRNAs as Diagnostic Biomarkers to Detect Specific Stages of Ovarian Cancer: A Comprehensive Meta-Analysis
Source: Cancers (Basel). 2024 Dec 16;16(24):4190. doi: 10.3390/cancers16244190 (PMC11674734; doi:10.3390/cancers16244190)
Supplement: Supplementary file 1 [file Table S1.pdf › Table S1.pdf]

RMIT Classification: Trusted

Table S1. Extracted data from the selected articles

#	Title of study	Authorship	Journal	Year	untry of st	Study type	Analysis method	Levels	Sample type	Age (med/mean)	# of partic	l # of con	of cancer p	FIGO stag	Type of control	Specificit	Sensitivit	miRNAs
1	Elevated levels of circulating microRNA	Kan et al	BMC Cancer	2012	Australia	Case-control	qPCR	Increased	Serum	NA	56	28	28	III-IV	Healthy control	46%	78%	miR200a, miR200b, miR200c
							qPCR	Increased	Serum	NA	56	28	28	III-IV	Healthy control	36%	86%	miR200a
							qPCR	Increased	Serum	NA	56	28	28	III-IV	Healthy control	36%	86%	miR200b
							qPCR	Increased	Serum	NA	56	28	28	III-IV	Healthy control	57%	71%	miR200c
2	Plasma miRNA as diagnositic and progr	Zheng et al	PLoS One	2013	China	Case-control	qPCR	Increased	Plasma	53 vs matched	204	70	134	I-IV	Healthy control	94%	30%	miR205
							qPCR	Decreased	Plasma	53 vs matched	204	70	134	I-IV	Healthy control	84%	67%	let7f
							qPCR	Increased and decreased	Plasma	53 vs matched	250	100	150	I-IV	Healthy control	82%	71%	miR205, let7f
							qPCR	Increased and decreased	Plasma	53 vs matched	125	100	25	I-II	Healthy control	82%	70%	miR205, let7f
3	Plasma microRNAs as novel biomarkers	Suryawanshi et	Clinical Cancer Rese	2013	USA	Case-control	qPCR	Increased	Plasma	47 vs matched	34	20	14	I-IV	Healthy control	85%	86%	miR16, miR21, miR191
							qPCR	Increased	Plasma	47 vs matched	41	20	21	I-IV	Healthy control	55%	90%	miR16, miR21, miR191
4	Serum microRNA-92 expression in pati	Guo et al	Journal of Internatio	2013	China	Case-control	qPCR	Increased	Serum	45 vs matched	100	50	50	I-IV	Healthy control	75%	80%	miR92
5	MicroRNA-200c and microRNA-141 as	Gao and Wu	Tumour Biology	2015	China	Case-control	qPCR	Increased	Serum	NA	104	50	54	I-II	Healthy control	50%	93%	miR200c
							qPCR	Increased	Serum	NA	104	50	54	I-II	Healthy control	50%	59%	miR141
							qPCR	Increased	Serum	NA	70	50	20	III-IV	Healthy control	50%	70%	miR200c
							qPCR	Increased	Serum	NA	70	50	20	III-IV	Healthy control	50%	85%	miR141
							qPCR	Increased	Serum	NA	143	50	93	I-IV	Healthy control	70%	72%	miR200c
							qPCR	Increased	Serum	NA	143	50	93	I-IV	Healthy control	72%	69%	miR141
6	Diagnostic and prognostic potential of	Meng et al	British Jurnal of Canc	2015	Germany	Case-control	qPCR	Increased	Serum	60 vs matched	246	66	180	I-IV	Healthy control	74%	53%	miR7
							qPCR	Decreased	Serum	60 vs matched	246	66	180	I-IV	Healthy control	64%	92%	miR25
							qPCR	Decreased	Serum	60 vs matched	246	66	180	I-IV	Healthy control	63%	88%	miR93
							qPCR	increased	Serum	60 vs matched	246	66	180	I-IV	Healthy control	96%	59%	miR429
							qPCR	Increased and decreased	Serum	60 vs matched	246	66	180	I-IV	Healthy control	92%	93%	miR7, miR429, miR25, miR93
7	Combined measurement of miRNA-18	Liang et al	European Journal of	2020	China	Case-control	qPCR	Increased	Serum	50 vs matched	151	50	101	I-IV	Benign control	80%	72%	miR183
							qPCR	Increased	Serum	50 vs matched	151	50	101	I-IV	Benign control	27%	91%	miR182
							qPCR	Increased	Serum	50 vs matched	151	50	101	I-IV	Benign control	85%	97%	CA125, HE4, miR183
							qPCR	Increased	Serum	50 vs matched	148	50	98	I-IV	Benign control	82%	94%	CA125, HE4, miR183
							qPCR	Increased	Serum	50 vs matched	151	50	101	I-IV	Benign control	70%	69%	miR205
8	Expression of serum miR-200a, miR-2	Zuberi et al	Cinical and Translati	2015	India	Case-control	qPCR	Increased	Serum	45 vs matched	140	70	70	I-IV	Healthy control	73%	83%	miR200a, miR200b, miR200c
9	Circulating Cell-Free miR-373, miR-20	Meng et al	Advancesin Experimental Medicine and Biology	2016	Germany	Case-control	qPCR	Increased	Serum	56 vs matched	80	20	60	I-IV	Benign control	100%	83%	miR200a, miR200b, miR200c
							qPCR	Increased	Serum	56 vs matched	80	20	60	I-IV	Benign control	90%	67%	miR200a
							qPCR	Increased	Serum	56 vs matched	80	20	60	I-IV	Benign control	90%	93%	miR200b
							qPCR	Increased	Serum	56 vs matched	80	20	60	I-IV	Benign control	85%	86%	miR200c
10	Diagnostic and prognostic relevance of	Meng et al	Oncotarget	2016	Germany	Case-control	qPCR	Increased	Serum	60 vs 46 vs 56	183	20	163	I-IV	Benign control	90%	88%	miR200a, miR200b, miR200c
							qPCR	Increased	Serum	60 vs 46 vs 56	183	20	163	I-IV	Benign control	100%	31%	miR200c
							qPCR	Increased	Serum	60 vs 46 vs 56	183	20	163	I-IV	Benign control	100%	53%	miR200b
							qPCR	Increased	Serum	60 vs 46 vs 56	183	20	163	I-IV	Benign control	90%	84%	miR200a
11	miR-199a and its association with clini	Zuberi et al	Tumour Biology	2016	India	Case-control	qPCR	Decreased	Serum	45 vs matched	140	70	70	I-IV	Healthy control	95%	69%	miR199a
12	Utility of serum miR-125b as a diagnos	Zuberi et al	PLoS One	2016	India	Case-control	qPCR	Increased	Serum	41 vs matched	140	70	70	I-IV	Healthy control	77%	62%	miR125b
13	A pilot study of circulating MicroRNA-1	Zhu et al	International Journal	2017	China	Case-control	qPCR	Increased	Serum	54 vs matched	189	54	135	I-IV	Benign control	69%	76%	miR125b
14	Circulating miRNA landscape identifie	Todeschini et a	Cancer Letters	2017	Italy	Case-control	qPCR	Increased	Serum	58 vs 52	162	52	110	III-IV	Healthy control	71%	93%	miR1246
							qPCR	Increased	Serum	58 vs 52	162	52	110	III-IV	Healthy control	86%	44%	miR595
							qPCR	Increased	Serum	58 vs 52	162	52	110	III-IV	Healthy control	69%	91%	miR2278
							qPCR	Increased	Serum	58 vs 52	71	13	58	III-IV	Healthy control	100%	77%	miR1246
							qPCR	Increased	Serum	58 vs 52	71	13	58	III-IV	Healthy control	100%	48%	miR595
							qPCR	Increased	Serum	58 vs 52	71	13	58	III-IV	Healthy control	100%	31%	miR2278
							qPCR	Increased	Serum	58 vs 52	162	52	110	III-IV	Healthy control	92%	71%	miR1246, miR595, miR2278
							qPCR	Increased	Serum	58 vs 52	71	13	58	III-IV	Healthy control	74%	100%	miR1246, miR595, miR2278
15	A combination of circulating miRNAs fo	Yokoi et al	Oncotarget	2017	Japan	Case-control	qPCR	Increased	Serum	51 vs matched	108	43	65	I-II	Benign control	83%	86%	miR200a, miR766, miR26a, miR142, let7d, miR130b and miR328
							qPCR	Increased	Serum	51 vs matched	218	63	155	I-IV	Healthy control	92%	91%	miR200a, miR766, miR26a, miR142, let7d, miR130b, miR328, and miR374
							qPCR	Increased	Serum	51 vs matched	218	63	155	I-IV	Healthy control	95%	98%	CA125, miR200a, miR766, miR26a, miR142, let7d and miR328
							qPCR	Increased	Serum	51 vs matched	30	15	15	I-II	Healthy control	53%	87%	miR142
							qPCR	Increased	Serum	51 vs matched	30	15	15	I-II	Healthy control	53%	80%	miR26a
							qPCR	Increased	Serum	51 vs matched	30	15	15	I-II	Healthy control	53%	80%	let7d
							qPCR	Increased	Serum	51 vs matched	30	15	15	I-II	Healthy control	53%	80%	miR374a
							qPCR	Increased	Serum	51 vs matched	30	15	15	I-II	Healthy control	53%	80%	miR766
							qPCR	Increased	Serum	51 vs matched	30	15	15	I-II	Healthy control	53%	80%	miR200a
							qPCR	Increased	Serum	51 vs matched	30	15	15	I-II	Healthy control	53%	80%	miR328
							qPCR	Increased	Serum	51 vs matched	30	15	15	III-IV	Healthy control	53%	80%	miR130b
							qPCR	Increased	Serum	51 vs matched	30	15	15	III-IV	Healthy control	53%	80%	miR142
							qPCR	Increased	Serum	51 vs matched	30	15	15	III-IV	Healthy control	53%	87%	miR26a
							qPCR	Increased	Serum	51 vs matched	30	15	15	III-IV	Healthy control	53%	73%	let7d
							qPCR	Increased	Serum	51 vs matched	30	15	15	III-IV	Healthy control	53%	87%	miR374a
							qPCR	Increased	Serum	51 vs matched	30	15	15	III-IV	Healthy control	53%	53%	miR766
							qPCR	Increased	Serum	51 vs matched	30	15	15	III-IV	Healthy control	53%	87%	miR200a
							qPCR	Increased	Serum	51 vs matched	30	15	15	III-IV	Healthy control	53%	40%	miR328
							qPCR	Increased	Serum	51 vs matched	45	15	30	I-IV	Healthy control	53%	81%	miR130b
							qPCR	Increased	Serum	51 vs matched	45	15	30	I-IV	Healthy control	53%	83%	miR142
							qPCR	Increased	Serum	51 vs matched	45	15	30	I-IV	Healthy control	53%	83%	miR26a
							qPCR	Increased	Serum	51 vs matched	45	15	30	I-IV	Healthy control	53%	77%	let7d
							qPCR	Increased	Serum	51 vs matched	45	15	30	I-IV	Healthy control	53%	83%	miR374a
							qPCR	Increased	Serum	51 vs matched	45	15	30	I-IV	Healthy control	53%	67%	miR766
							qPCR	Increased	Serum	51 vs matched	45	15	30	I-IV	Healthy control	53%	83%	miR200a
							qPCR	Increased	Serum	51 vs matched	45	15	30	I-IV	Healthy control	53%	60%	miR328
							qPCR	Increased	Serum	51 vs matched	45	15	30	I-IV	Healthy control	53%	81%	miR130b
16	Diagnostic potential for a serum miRNA	Elias et al	eLife	2017	USA	Case-control	sequencing and q	Increased and decreased	Serum	55 vs matched	160	70	90	I-IV	Healthy control	100%	75%	14 miRNAs
							sequencing and q	Increased and decreased	Serum	55 vs matched	51	29	22	I-IV	Benign control	78%	91%	miR29a, miR92a, miR200c, miR320c, miR335, miR450b, and miR1307
17	Exosomal miR-1290 is a potential biom	Kobayashi et a	Journal of Ovarian Re	2018	Japan	Case-control	qPCR	Increased	Serum	58 vs 34	70	13	30	I-IV	Healthy control	85%	63%	miR1290
18	Exosomal microRNAs as tumour marke	Pan et al	Molecular Oncology	2018	Germany	Case-control	microarray/qPCR	Increased	Plasma	60 vs 56	135	29	106	I-IV	Healthy control	82%	61%	miR21
							microarray/qPCR	Increased	Plasma	60 vs 56	135	29	106	I-IV	Healthy control	73%	62%	miR100
							microarray/qPCR	Increased	Plasma	60 vs 56	135	29	106	I-IV	Healthy control	86%	64%	miR200b
							microarray/qPCR	Increased	Plasma	60 vs 56	135	29	106	I-IV	Healthy control	69%	56%	miR320
19	Integrated extracellular microRNA prof	Yokoi et al	Nature Communicati	2018	Japan	Case-control	qPCR	Increased and decreased	Serum	57 vs matched	223	63	160	I-IV	Benign control and healthy	80%	64%	miR663b, miR4730, miR642a, miR658, miR486, miR1246, miR1207, miR4419b, miR6124
							qPCR	Increased and decreased	Serum	57 vs matched	307	147	160	I-IV	Benign control and healthy	91%	82%	miR663b, miR4730, miR642a, miR658, miR486, miR1246, miR1207, miR4419b, miR6124
20	Exosomal miR-99a-50 is elevated in se	Yoshimura et a	BMC Cancer	2018	Japan	Case-control	qPCR	Increased	Serum	59 vs 52 vs 35	108	20	88	I-IV	Healthy control	75%	85%	miR99a
21	Diagnostic Model of Serum miR-193a-5	Ren et al	Pathology and Oncol	2018	China	Case-control	qPCR	Decreased	Serum	56 vs 30 vs 54	85	40	45	I-IV	Healthy control	97%	93%	miR193a
							qPCR	Decreased	Serum	56 vs 30 vs 54	75	30	45	I-IV	Benign control	60%	87%	miR193a
							qPCR	Increased and decreased	Serum	56 vs 30 vs 54	85	40	45	I-IV	Healthy control	67%	73%	miR193a, HE4 and CA125
22	Serum MicroRNA-21 Negatively Relate	Mahmoud et al	Asian Pac J ournal of	2018	Egypt	Case-control	qPCR	Increased	Serum	55 vs matched	90	30	60	I-IV	Healthy control	80%	97%	miR21
23	Serum exosomal miRNA-145 and miRN	Kim et al	Journal of Cancer	2019	Korea	Case-control	qPCR	Increased	Serum	51 vs matched	68	20	48	I-IV	Benign control	75%	92%	miR145
							qPCR	Increased	Serum	51 vs matched	68	20	48	I-IV	Benign control	90%	73%	miR200c
							qPCR	Increased	Serum	51 vs matched	68	20	48	I-IV	Benign control	60%	98%	CA125, miR145
							qPCR	Increased	Serum	51 vs matched	68	20	48	I-IV	Benign control	70%	94%	CA125, miR200c
							qPCR	Increased	Serum	51 vs matched	68	20	48	I-IV	Benign control	65%	94%	miR145, miR200c
							qPCR	Increased	Serum	51 vs matched	68	20	48	I-IV	Benign control	55%	100%	CA125, miR145, miR200c
24	Diagnostic plasma miRNA-profiles for	Oliviera et al	PLoS One	2019	Denmark	Case-control	qPCR	Increased	Serum	66 vs 51	190	95	95	I-IV	Benign control	95%	80%	miR200c, miR221
25	Five serum microRNAs for detection an	Wang et al	European Journal of	2019	China	Case-control	qPCR	Increased	Serum	45 vs matched	160	80	80	I-IV	Healthy control	52%	81%	miR200c
							qPCR	Increased	Serum	45 vs matched	160	80	80	I-IV	Healthy control	63%	72%	miR346
							qPCR	Increased	Serum	45 vs matched	160	80	80	I-IV	Healthy control	53%	89%	miR127
							qPCR	Increased	Serum	45 vs matched	160	80	80	I-IV	Healthy control	47%	85%	miR143
							qPCR	Increased	Serum	45 vs matched	160	80	80	I-IV	Healthy control	53%	76%	miR205
26	Circulating epithelial-mesenchymal tra	Marton et al	Journal of Biotechnology	2019	Hungary	Case-control	qPCR	Increased	Plasma	57 vs 56	100	72	28	I-IV	Benign control and healthy	86%	81%	miR200a
							qPCR	Increased	Plasma	57 vs 56	100	72	28	I-IV	Benign control and healthy	68%	83%	miR200b
							qPCR	Increased	Plasma	57 vs 56	100	72	28	I-IV	Benign control and healthy	71%	82%	miR200c
							qPCR	Increased	Plasma	57 vs 56	100	72	28	I-IV	Benign control and healthy	86%	82%	miR141
							qPCR	Increased	Plasma	57 vs 56	100	72	28	I-IV	Benign control and healthy	86%	74%	miR429
							qPCR	Increased	Plasma	57 vs 56	100	72	28	I-IV	Benign control and healthy	82%	81%	miR203a
							qPCR	Increased	Plasma	57 vs 56	100	72	28	I-IV	Benign control and healthy	43%	70%	miR34a
							qPCR	Increased	Plasma	57 vs 56	100	72	28	I-IV	Benign control and healthy	71%	74%	miR34b
27	Role of a novel circulatory RNA-based	El Shal et al	IUBMB Life	2019	Egypt	Case-Control	qPCR	Decreased	Serum	52 vs 48	137	87	50	I-IV	Benign control and healthy	70%	84%	miR361
28	Serum microRNA-145 as a novel bioma	Liang et al	Tumour Biology	2015	China	Case-control	qPCR	Decreased	Serum	58 vs matched	229	135	94	I-IV	Healthy control	50%	93%	miR145
							qPCR	Decreased	Serum	58 vs matched	145	51	94	I-IV	Benign control	51%	84%	miR145
29	Expression and clinical significance of	Chen et al	Oncologie	2020	China	Case-control	qPCR	Decreased	Serum	48 vs matched	104	30	74	I-IV	Benign control	86%	57%	miR152
30	Serum miR-125b levels associated wit	Chen et al	Bioengineered	2020	China	Case-control	qPCR	Decreased	Serum	51 vs matched	291	139	152	I-IV	Benign control and healthy	96%	84%	miR125b, CA125
							qPCR	Decreased	Serum	51 vs matched	301	149	152	I-IV	Benign control and healthy	42%	76%	miR125b
31	The Promising Signatures of Circulating	Zuberi et al	Microrna	2020	India	Case-control	qPCR	Decreased	Serum	45 vs matched	103	70	33	I-II	Healthy control	96%	94%	miR145
							qPCR	Decreased	Serum	45 vs matched	107	70	37	III-IV	Healthy control	96%	92%	miR145
							qPCR	Decreased	Serum	45 vs matched	140	70	70	I-IV	Healthy control	96%	69%	miR145
32	Multi-analytical test based on serum m	Cirillo et al	PLoS One	2021	Italy	Case-control	ddPCR	Increased	Serum	61 vs matched	115	68	47	I-IV	Healthy control	88%	72%	miR320b, miR141
							ddPCR	Increased	Serum	61 vs matched	115	68	47	I-IV	Healthy control	100%	76%	miR320b, miR141, HE4, CA125
							ddPCR	Increased	Serum	61 vs matched	88	68	20	I-II	Healthy control	88%	75%	miR320b, miR141
							ddPCR	Increased	Serum	61 vs matched	88	68	20	I-II	Healthy control	100%	70%	miR320b, miR141, HE4, CA125
							ddPCR	Increased	Serum	61 vs matched	95	68	27	III-IV	Healthy control	88%	70%	miR320b, miR141
							ddPCR	Increased	Serum	61 vs matched	95	68	27	III-IV	Healthy control	100%	80%	miR320b, miR141, HE4, CA125
							ddPCR	Increased	Serum	62 vs matched	62	37	25	I-IV	Healthy control	73%	68%	miR320b, miR141
							ddPCR	Increased	Serum	62 vs matched	62	37	25	I-IV	Healthy control	100%	87%	miR320b, miR141, HE4, CA125
							ddPCR	Increased	Serum	62 vs matched	47	37	10	I-II	Healthy control	70%	80%	miR320b, miR141
							ddPCR	Increased	Serum	62 vs matched	47	37	10	I-II	Healthy control	100%	89%	miR320b, miR141, HE4, CA125
							ddPCR	Increased	Serum	62 vs matched	52	37	15	III-IV	Healthy control	70%	60%	miR320b, miR141
							ddPCR	Increased	Serum	62 vs matched	52	37	15	III-IV	Healthy control	100%	86%	miR320b, miR141, HE4, CA125
33	Evaluation of diagnositc potential of ep	Kumar et al	Fontiers in Oncology	2021	India	Case-control	qPCR	Increased	Serum	45 vs matched	65	21	44	III-IV	Healthy control	73%	86%	miR205
							qPCR	Increased	Serum	45 vs matched	65	21	44	III-IV	Healthy control	68%	90%	miR200c
							qPCR	Increased	Serum	45 vs matched	65	21	44	III-IV	Healthy control	73%	93%	miR141
							qPCR	Increased	Serum	45 vs matched	65	30	35	I-II	Healthy control	73%	81%	miR205
							qPCR	Increased	Serum	45 vs matched	65	30	35	I-II	Healthy control	80%	71%	miR200c
							qPCR	Increased	Serum	45 vs matched	65	30	35	I-II	Healthy control	80%	81%	miR141
34	Preliminary evaluation of miR-1307-3p	Hashimoto et a	Heliyon	2021	Japan	Case-Control	microRNA chip	Increased	Serum	57 vs 59	20	14	6	I-IV	Healthy control	100%	100%	miR1307
							qPCR	Increased	Serum	57 vs 59	20	13	7	I-IV	Healthy control	85%	100%	miR1307
35	Circulating exosomal miRNA-1290 for	Jeon et al	Current ilsue in Mole	2022	Korea	Case-control	qPCR	Increased	Serum	52 vs matched	84	13	71	I-IV	Benign control	69%	79%	miR1290
36	Detection of plasma exosomal miRNA-	Zhu et al	Journal for Ovarian R	2022	China	Case-control	qPCR	Increased	Plasma	57 vs 49 vs 51	68	32	36	I-IV	Healthy control	78%	67%	miR205
							qPCR	Increased	Plasma	57 vs 49 vs 51	68	32	36	I-IV	Healthy control	83%	97%	miR205, CA125
							qPCR	Increased	Plasma	57 vs 49 vs 51	68	32	36	I-IV	Healthy control	69%	97%	miR205, HE4
							qPCR	Increased	Plasma	57 vs 49 vs 51	68	32	36	I-IV	Healthy control	86%	100%	miR205, CA125, HE4
37	Transcriptomic analysis of patient plas	Hannan et al	Gynecologic Oncolog	2022	Australia	Case-Control	NGS and qPCR	Increased	Plasma	63 vs 60	48	24	24	III-IV	Benign control	54%	92%	miR200c
							qPCR	Increased	Plasma	63 vs 60	29	14	15	III-IV	Benign Control	60%	71%	miR200c
38	Sensitivity and specificity of microRNA	Ali et al	PLoS One	2022	Egypt	Case-Control	qPCR	Increased	Serum	45 vs 40	120	30	90	I-IV	Benign control and healthy	58%	98%	miR204
39	A Novel Blood-Based microRNA Diagno	Zhang and Hu	Cancers (Basel)	2022	USA	Case-Control	qPCR	Increased	Serum	57 vs matched	3092	2759	333	I-IV	Healthy control	99%	68%	miR5100, miR1343, miR1290, and miR4787
							qPCR	Increased	Serum	57 vs matched	2087	1754	333	I-IV	Healthy control	95%	90%	miR5100, miR1343, miR1290, and miR4787
40	Plasma exosomal miR-320d, miR-4479	Wang et al	Frontiers in Oncolog	2022	China	Case-Control	qPCR	Decreased	Plasma	60 vs matched	329	168	161	I-IV	Healthy control	92%	35%	miR320d
							qPCR	Decreased	Plasma	60 vs matched	329	168	161	I-IV	Healthy control	71%	76%	miR4479
							qPCR	Decreased	Plasma	60 vs matched	329	168	161	I-IV	Healthy control	54%	75%	miR6763
							qPCR	Decreased	Plasma	60 vs matched	329	168	161	I-IV	Healthy control	72%	73%	miR6763
							qPCR	Decreased	Plasma	60 vs matched	195	34	161	I-IV	Benign control	71%	71%	miR320d
							qPCR	Decreased	Plasma	60 vs matched	195	34	161	I-IV	Benign control	77%	58%	miR4479
							qPCR	Decreased	Plasma	60 vs matched	195	34	161	I-IV	Benign control	91%	54%	miR6763
							qPCR	Decreased	Plasma	60 vs matched	195	34	161	I-IV	Benign control	82%	66%	miR6763
41	Plasma exosomal miR-1260a, miR-797	Chen etal	Future Oncology	2022	China	Case-control	qPCR	Decreased	Plasma	59 vs matched	270	136	134	I-IV	Healthy control	74%	66%	miR1260a
							qPCR	Decreased	Plasma	59 vs matched	270	136	134	I-IV	Healthy control	82%	49%	miR7977
							qPCR	Decreased	Plasma	59 vs matched	270	136	134	I-IV	Healthy control	87%	74%	miR192
							qPCR	Decreased	Plasma	59 vs matched	270	136	134	I-IV	Healthy control	89%	71%	miR1260a, miR7977, miR192
							qPCR	Decreased	Plasma	59 vs matched	149	136	13	I-II	Healthy control	50%	85%	miR1260a
							qPCR	Decreased	Plasma	59 vs matched	148	135	13	I-II	Healthy control	50%	69%	miR7977
							qPCR	Decreased	Plasma	59 vs matched	143	130	13	I-II	Healthy control	50%	69%	miR192
42	Evaluation of the Diagnostic Potential	Raiser et al	Anticancer Research	2023	Germany	Case-Control	qPCR	Increased	Serum	63 vs 60	76	28	48	I-IV	Benign control	61%	67%	miR21
							qPCR	Decreased	Serum	63 vs 60	76	28	48	I-IV	Benign control	50%	71%	miR1
43	Circulating cf-miRNA as a more approp	Gahlawat et al	Scientific Reports	2023	Germany	Case-Control	qPCR	Increased	Plasma	NA	125	53	72	I-IV	Benign control and healthy	81%	66%	miR200c and miR141
44	Identification of serum miR-1246 and r	Niemira et al	Scientific Reports	2023	Poland	Case-Control	nanostring	Increased and decreased	Serum	61 vs 58	49	21	28	I-IV	Healthy control	95%	96%	miR1246 and miR150
							nanostring	Increased and decreased	Serum	59 vs 61	21	13	8	I-IV	Healthy control	92%	100%	miR1246 and miR150
							nanostring	Increased and decreased	Serum	61 vs 58	49	21	28	I-IV	Healthy control	95%	93%	miR1246 and miR144
							nanostring	Increased and decreased	Serum	59 vs 61	21	13	8	I-IV	Healthy control	88%	92%	miR1246 and miR144
							qPCR	Increased and decreased	Serum	61 vs 58	62	34	28	I-IV	Healthy control	94%	96%	miR1246 and miR150
							qPCR	Increased and decreased	Serum	59 vs 61	26	12	14	I-IV	Healthy control	92%	100%	miR1246 and miR150
							qPCR	Increased and decreased	Serum	NA	448	224	224	I-IV	Healthy control	87%	81%	miR1246 and miR150
							qPCR	Increased and decreased	Serum	NA	192	96	96	I-IV	Healthy control	88%	82%	miR1246 and miR150
							qPCR	Increased and decreased	Serum	NA	400	320	80	I-II	Healthy control	78%	87%	miR1246 and miR150
							qPCR	Increased and decreased	Serum	NA	350	320	30	I-II	Healthy control	87%	93%	miR1246 and miR150
							qPCR	Increased and decreased	Serum	NA	432	320	112	III-IV	Healthy control	87%	77%	miR1246 and miR150
							qPCR	Increased and decreased	Serum	NA	352	320	32	III-IV	Healthy control	89%	75%	miR1246 and miR150
45	miR-146a-5p and miR-191-5p as novel	Takamizawa et	Molecular and Clinic	2023	Japan	Case-Control	Tagman array	Increased	Serum	54 vs 53	47	18	29	I-IV	Healthy control	50%	79%	miR146a
							Tagman array	Increased	Serum	54 vs 53	47	18	29	I-IV	Healthy control	50%	90%	miR191
46	Identifying Serum Small Extracellular V	Li et al	ACS Nano	2023	China	Case-Control	mirna sequencing	Increased	Serum	50 vs 50	29	13	16	I-IV	Benign control	92%	88%	miR1246, miR141, miR200a, miR200b, miR200c, miR203a, and miR-429
							mirna sequencing	Increased	Serum	50 vs 50	20	5	15	I-IV	Benign control	100%	87%	miR1246, miR141, miR200a, miR200b, miR200c, miR203a, and miR-429
							mirna sequencing	Increased	Serum	50 vs 50	30	15	15	I-IV	Benign control	93%	87%	miR1246, miR141, miR200a, miR200b, miR200c, miR203a, and miR-429
							qPCR	Increased	Serum	50 vs 50	35	16	19	I-IV	Benign control	63%	74%	miR1246
							qPCR	Increased	Serum	50 vs 50	35	16	19	I-IV	Benign control	56%	84%	miR141
							qPCR	Increased	Serum	50 vs 50	35	16	19	I-IV	Benign control	50%	95%	miR200a
							qPCR	Increased	Serum	50 vs 50	35	16	19	I-IV	Benign control	69%	84%	miR200b
							qPCR	Increased	Serum	50 vs 50	35	16	19	I-IV	Benign control	63%	74%	miR200c
							qPCR	Increased	Serum	50 vs 50	35	16	19	I-IV	Benign control	63%	74%	miR203a
							qPCR	Increased	Serum	50 vs 50	35	16	19	I-IV	Benign control	63%	89%	miR429
							qPCR	Increased	Serum	50 vs 50	79	33	46	I-IV	Benign control	85%	81%	miR429
							qPCR	Increased	Serum	50 vs 50	79	33	46	I-IV	Benign control	100%	77%	miR429, miR1246
							qPCR	Increased	Serum	50 vs 50	79	33	46	I-IV	Benign control	100%	82%	miR429, miR1246, miR200b
							qPCR	Increased	Serum	50 vs 50	79	33	46	I-IV	Benign control	100%	82%	miR429, miR1246, miR200b, miR200c
							qPCR	Increased	Serum	50 vs 50	79	33	46	I-IV	Benign control	100%	82%	miR429, miR1246, miR200b, miR200c, miR141
							qPCR	Increased	Serum	50 vs 50	79	33	46	I-IV	Benign control	100%	77%	miR429, miR1246, miR200b, miR200c, miR141, miR203
47	Metabolic pathways of potential miRNA	Gumusoglu-Ac	Oncology Letters	2023	Turkey	Case-Control	qPCR	Decreased	Serum	45 vs 44	20	10	10	I-IV	Healthy control	60%	58%	miR1909
							qPCR	Decreased	Serum	45 vs 44	20	10	10	I-IV	Healthy control	65%	64%	miR885
							qPCR	Decreased	Serum	45 vs 44	20	10	10	I-IV	Healthy control	61%	60%	let7d
48	Diagnostic value of plasma-derived exo	Yang et al	BMC Womens Health	2024	China	Case-Control	qPCR	Increased	Plasma	57 vs 57	130	52	78	I-IV	Healthy control	73%	83%	miR223
							qPCR	Increased	Plasma	57 vs 57	130	52	78	I-IV	Healthy control	87%	94%	miR223, CA125
							qPCR	Increased	Plasma	57 vs 57	118	40	78	I-IV	Benign control	73%	73%	miR223
							qPCR	Increased	Plasma	57 vs 57	118	40	78	I-IV	Benign control	78%	95%	miR223, CA125
							qPCR	Increased	Plasma	57 vs 57	91	52	39	I-II	Healthy control	73%	77%	miR-223
							qPCR	Increased	Plasma	57 vs 57	91	52	39	I-II	Healthy control	89%	97%	miR223, CA125
							qPCR	Increased	Plasma	57 vs 57	79	40	39	I-II	Benign control	50%	72%	miR-223
49	The evaluation of miR-1181 and miR-4	Minareci et al	Molecular Biology Re	2024	Turkey	Case-Control	qPCR	Increased	Serum	61 vs 61	135	66	69	I-IV	Healthy control	70%	72%	miR4314
							qPCR	Increased	Serum	61 vs 61	135	66	69	I-IV	Healthy control	73%	71%	miR1181
50	Aberrant miR-3135b and miR-1273g-3	Tuncer et al	Heliyon	2024	Turkey	Case-Control	qPCR	Decreased	Blood	49 vs matched	250	100	150	I-IV	Healthy control	62%	72%	miR3135b
							qPCR	Decreased	Blood	49 vs matched	250	100	150	I-IV	Healthy control	49%	58%	miR1273g
